# Augmented reality‐guided positioning system for radiotherapy patients

**DOI:** 10.1002/acm2.13516

**Published:** 2022-01-05

**Authors:** Chunying Li, Zhengda Lu, Mu He, Jianfeng Sui, Tao Lin, Kai Xie, Jiawei Sun, Xinye Ni

**Affiliations:** ^1^ Department of Radiotherapy, Changzhou Second People's Hospital Nanjing Medical University Changzhou China; ^2^ Laboratory of Medical Physics Center Nanjing Medical University, Jiangning District Nanjing China; ^3^ Changzhou Key Laboratory of Medical Physics Changzhou China

**Keywords:** augmented reality, position setup, position setup error, radiation therapy

## Abstract

In modern radiotherapy, error reduction in the patients’ daily setup error is important for achieving accuracy. In our study, we proposed a new approach for the development of an assist system for the radiotherapy position setup by using augmented reality (AR). We aimed to improve the accuracy of the position setup of patients undergoing radiotherapy and to evaluate the error of the position setup of patients who were diagnosed with head and neck cancer, and that of patients diagnosed with chest and abdomen cancer. We acquired the patient's simulation CT data for the three‐dimensional (3D) reconstruction of the external surface and organs. The AR tracking software detected the calibration module and loaded the 3D virtual model. The calibration module was aligned with the Linac isocenter by using room lasers. And then aligned the virtual cube with the calibration module to complete the calibration of the 3D virtual model and Linac isocenter. Then, the patient position setup was carried out, and point cloud registration was performed between the patient and the 3D virtual model, such the patient's posture was consistent with the 3D virtual model. Twenty patients diagnosed with head and neck cancer and 20 patients diagnosed with chest and abdomen cancer in the supine position setup were analyzed for the residual errors of the conventional laser and AR‐guided position setup. Results show that for patients diagnosed with head and neck cancer, the difference between the two positioning methods was not statistically significant (*P* > 0.05). For patients diagnosed with chest and abdomen cancer, the residual errors of the two positioning methods in the superior and inferior direction and anterior and posterior direction were statistically significant (*t* = −5.80, −4.98, *P* < 0.05). The residual errors in the three rotation directions were statistically significant (*t* = −2.29 to −3.22, *P* < 0.05). The experimental results showed that the AR technology can effectively assist in the position setup of patients undergoing radiotherapy, significantly reduce the position setup errors in patients diagnosed with chest and abdomen cancer, and improve the accuracy of radiotherapy.

## 1 INTRODUCTION

In modern radiotherapy, reduction of patients’ daily position setup error is important for accurate radiotherapy. Commonly used positioning techniques in radiotherapy include electronic portal imaging device (EPID), cone beam CT (CBCT), magnetic resonance guidance, surface‐guided radiation therapy (SGRT), and electromagnetic navigation technology. As an earlier image guidance technology, EPID can only obtain two‐dimensional (2D) anatomical structure information of patients with low accuracy and difficult to correct the rotation error of patients.^1^ CBCT is the most widely used image guidance technology. It can provide three‐dimensional (3D) anatomical structure information of patients, and it is easy to register with planning CT images. However, the radiation dose caused by CBCT scan cannot be ignored, and a large number of CBCT‐assisted position setup may lead to a significant increase in the incidence of secondary cancer in patients.^2^ Magnetic resonance guidance has advantages, such as high soft tissue resolution and no ionizing radiation but has disadvantages, such as high equipment cost, long imaging time,^3^ and no real‐time monitoring. SGRT projects a speckle pattern on the patient by using optical light and cameras with separate camera pods are used to detect the pattern and map the patient's surface.^4^, ^5^ The surface matching algorithm is used to calculate the real‐time error between the patient's current position and the reference body surface.^6^ The combination of SGRT and deep inspiratory breath hold in the treatment of left breast cancer can effectively reduce cardiac radiation exposure.^7^ However, the registration of optical body surface lacks credibility in the superior and inferior direction. After radiotherapy, skin pigmentation and changes in patients’ facial expressions will also affect the position setup correction of the SGRT system.^8^ Calypso electromagnetic navigation was originally used for prostate localization.^9^ The electromagnetic signal feedback from the implanted transponder is detected by an electromagnetic array, and the treatment target position is measured in the 3D space to track the target area. However, it involves an invasive operation,^10^ and the transponder is prone to deviation or non‐response problems.^11^

Augmented reality (AR) is a technology developed on the basis of traditional virtual reality. It superimposes computer‐generated virtual objects into the real environment, which is perceived by human senses and allows interactive operation. AR has been studied and used in surgery, where preoperative data are superimposed on the surgical site to enhance the perception of the physical environment.^12^ The experiments of Jud^13^ et al. and Porpiglia^14^ et al. proved that in surgery, the application of AR in surgery can significantly improve success rates. AR is completed through the following steps. First, the camera must obtain the real scene information and analyze the real scene and camera position information. Second, virtual objects need to be generated. Third, the affine transformation of the virtual object to the camera's view plane is calculated in accordance with the position information of the camera relative to the real scene. Finally, virtual objects are drawn on the view plane in accordance with affine transformation.

Our study aimed to develop a radiotherapy position setup guidance system based on AR technology and analyze the position setup errors assisted by AR for patients diagnosed with head and neck cancer and patients diagnosed with chest and abdomen cancer to explore the feasibility of using AR technology in improving the position setup accuracy of radiotherapy.

## 2 MATERIALS AND METHODS

### 2.1 Case data

This study included 40 patients, including 20 head and neck patients in the G1 group and 20 supine chest and abdomen cancer patients in the G2 group. Patients underwent radiotherapy with a linac accelerator (Elekta Infinity, Sweden). In the conventional laser position setup (LPT), fixed room lasers were used to align the patient's tattoo, whereas in AR positioning (ARPT), the room lasers were applied to align the patient's tattoo first, and then the patient's position setup was adjusted in accordance with the simulation CT position setup presented by the AR technology. CBCT images of the patients were collected after each LPT and ARPT, and automatic registration was performed based on bone tissue, which was confirmed and reviewed by experienced oncologists. The ARPT and LPT residual errors of each time were recorded using CBCT registration results as the gold standard. Forty patients were treated using LPT and ARPT alignment once a week, and data were collected for 3 weeks, resulting in a total of 120 sets of radiotherapy positioning residual error data.

### 2.2 System layout

**FIGURE 1:**
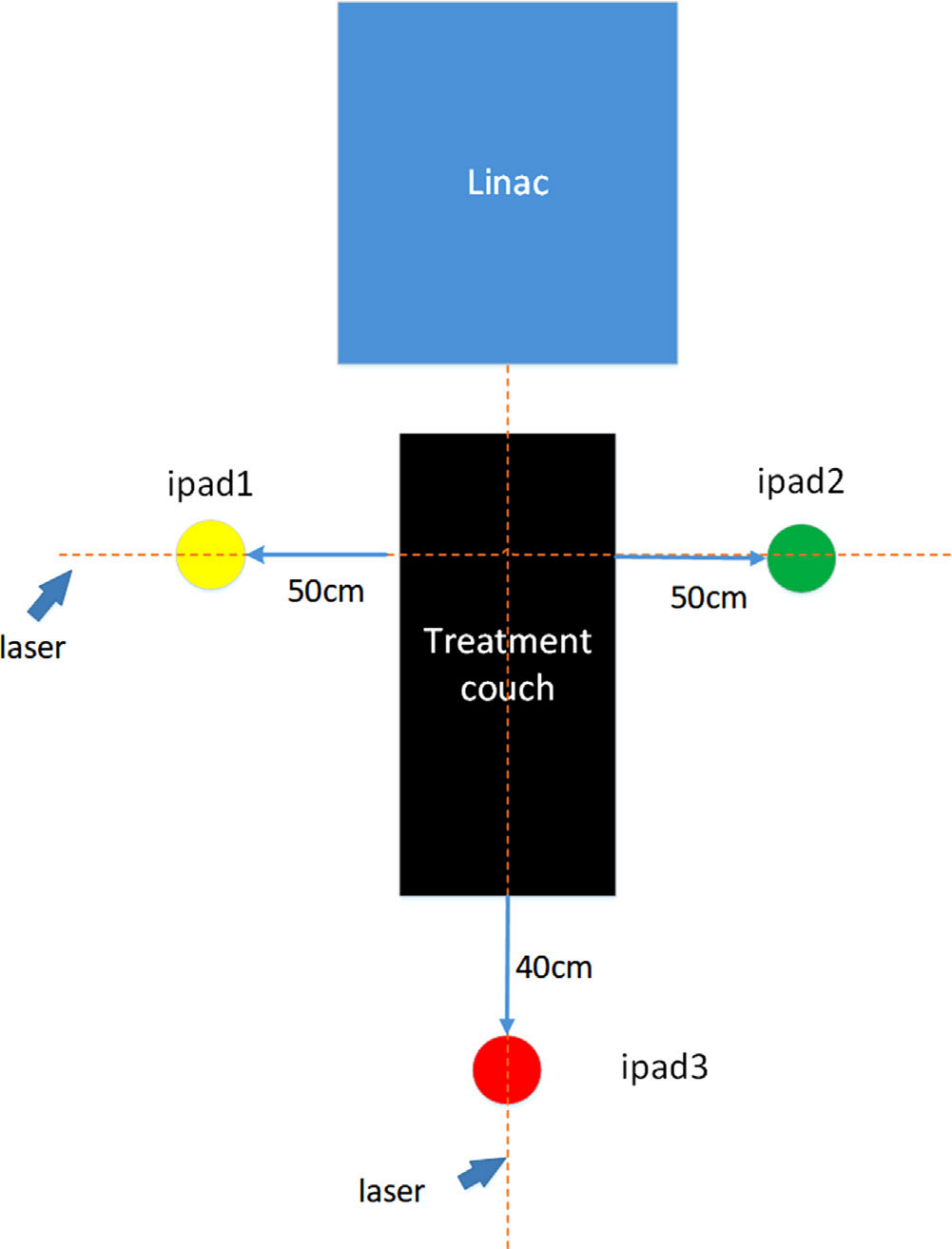
System layout

The system layout of AR‐guided radiotherapy is composed of three tablet devices (iPad Pro tablet 12.9 inch) and three iPad stands. Three iPads were fixed in the position shown in Figure 1, and three iPads were placed on both sides and at the end of the treatment couch. The heights of the iPad cameras on both sides of the treatment couch were horizontal to the room lasers, and the fore‐and‐aft direction position were consistent with the room lasers, and the left‐and‐right direction position were maintained at a distance of 50 cm from the treatment couch. The height of iPad camera at the end of the treatment couch was 60 cm higher than that of the room lasers, and the left‐and‐right direction position was consistent with the room lasers, and the fore‐and‐aft direction position was maintained at a distance of 40 cm from the end of the treatment couch. Multipeer‐connectivity near‐field communication technology was used for online communication to realize the real‐time sharing of environmental information and data.

### 2.3 3D modeling

The patients were scanned with CT (Somatom Force, Siemens, Germany) with a tube voltage of 120 kV. The tube current for patients with head and neck cancers was 250 mA , that for patients diagnosed with chest cancer was 200 mA, and that for patients diagnosed with abdominal cancer was 300 mA. The thickness of CT scanning slice was 5 mm and reconstruction increment was 2–3 mm. The simulation CT data were imported into the treatment planning system (TPS) for planning design. The structure file of the planning CT dataset was imported into a medical image processing software (3D Slicer version 4.11 National Institutes of Health) to conduct the 3D modeling of DICOM‐RT data and obtain a 3D virtual model.

### 2.4 Principles of AR simulation

**FIGURE 2:**
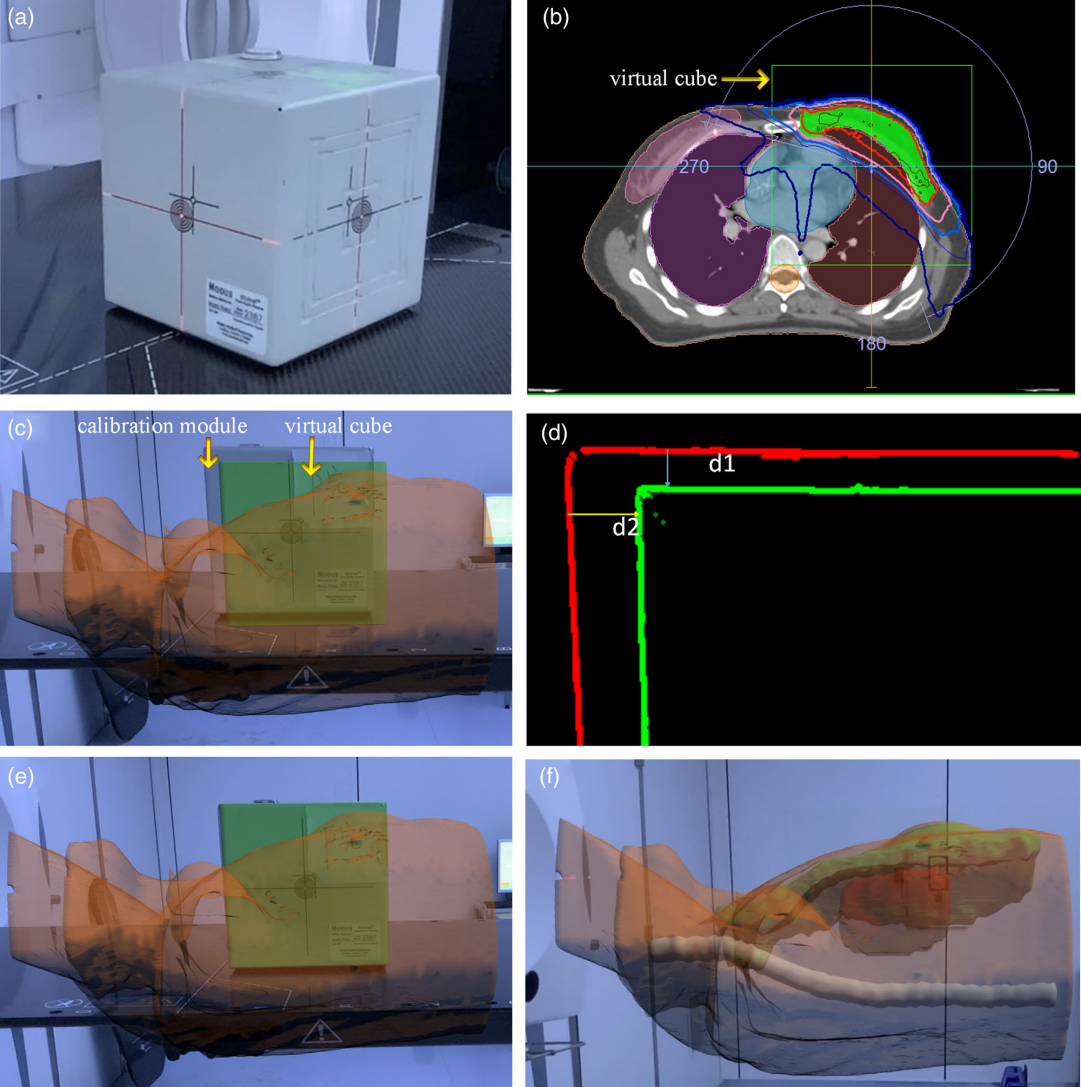
Isocentric calibration of the three‐dimensional (3D) virtual model (with breast patient as an example): (a) calibration module; (b) cross‐section of the human body, with the yellow arrow indicating the cross‐section of the virtual cube; (c) treatment couch side view. The calibration module is identified, and the 3D virtual model is loaded; (d) treatment couch side view. The contour distance between the calibration module and the virtual cube is *d*1, *d*2; (e) treatment couch side view. The virtual cube is aligned with the calibration module in all directions; (f) treatment couch side view. The calibration module is removed, and some normal tissues, planning target volume (PTV), and PTV (CTV), are displayed

The 3D virtual model was imported into Unity3D software, and ARKit was used as the underlying SDK to build an AR interactive system. The visual‐inertial odometry (VIO) technology was applied to combine the data from the gyroscope, accelerometer, LIDAR sensor, and other motion sensors of mobile devices with the image data collected by the camera for tracking and positioning. Thus, the 3D virtual model could be integrated with real environment. In this study, the calibration module shown in Figure 2a was scanned as the reference object, and its spatial characteristic information was recorded in advance. When the system was used for 3D object recognition and positioning, the feature points obtained by the camera were compared with the pre‐recorded feature points of the reference object. The positioning of the camera and the pose relative to the reference object was determined according to the coordinates of the corresponding feature points. Then, the mapping relationship between the virtual world and the real world was constructed by integrating the motion sensor information. The virtual object was rendered in accordance with the mapping relationship (Figure 2c) to complete the integration of the 3D virtual model and the real world. Key technologies included feature point extraction and matching and camera pose estimation.

#### 2.4.1 Feature point extraction and matching

The feature points of each frame were extracted and matched, based on which the camera motion and the location of the feature points were estimated roughly. Feature extraction mainly used VIO to obtain points with considerable differences in light and shade, color, and gray level in the video images. At the same time, combined with the LiDAR sensor of the device, the depth information of the feature points was obtained, as well as the 3D coordinates of the feature points. The main processes were FAST corner extraction and BRIEF descriptor creation. The scale invariance and rotation invariance were guaranteed by constructing image pyramid and intensity centroid method, respectively, to reduce the influence of scale and rotation changes on feature point detection. After the feature points were extracted, a BRIEF description of each feature point was made with the method of binary expression, was composed of many 1 and 0. Made a circle with the characteristic point as the center and *d* as the radius, selected *n* pairs of points *P*_1_(*A*,*B*), *P*_2_(*A*,*B*),…,*P_n_* (*A*,*B*) in the circle, and carried out τ operation(Equation (1)) on each pair of points, and obtained two descriptor vectors as shown in Equations (2) and (3). After the feature point was extracted, feature point matching was performed. The Hamming distance of the descriptor was calculated using Equation (4). The smaller *D* was, the higher the similarity between the two features, and the higher the matching degree of feature points.

(1) $$\tau \left(P;A,B\right)=\left\{\begin{array}{c} 1,P_{A}\geq P_{B}\\ 0,P_{A}<P_{B} \end{array}\right.,$$

where $P_{A}$ represents the gray level of point *A*, and $P_{B}$ represents the gray level of point *B*.

(2) $$\mathrm{brie}f_{1}=\left\{x_{1},x_{2},\text{…},x_{i}\;,\text{…},x_{n}\right\}$$

(3) $$\mathrm{brie}f_{2}=\left\{y_{1},y_{2},\text{…},y_{i},\text{…},y_{n}\right\},\;$$

where *n* is the total number of describing sub‐elements, and $x_{i}\;$and $y_{i}\;$are the size relations of two random pixels near the feature points, with values of 0 or 1.

(4) $$D\left(\mathrm{brie}f_{1},\;\mathrm{brie}f_{2}\right)=\sum_{i=1}^{n}\sqrt{x_{i}^{2}-y_{i}^{2}},$$

where $x_{i}$ is the ith element of $\mathrm{brie}f_{1}$, $y_{i}\;$is the ith element of $\mathrm{brie}f_{2}$, and *n* is the total number of datasets.

#### 2.4.2 Camera pose estimation

Camera pose estimation solved the problem of camera positioning and spatial map construction. After the feature points obtained by the system were accurately matched with the feature points of the reference object, the pose of the camera was estimated according to the pair of feature points, and the translation matrix *T* and rotation matrix *R* of the camera position relative to the scanning of the reference object were calculated. The 3D point set after matching is shown in Equations (5) and (6). A rotation matrix *R* and a shift vector *T* were sought to make Equation (7) always hold true. The least‐squares problem was constructed as shown in Equation (8) to solve *R* and *T* satisfying Equation (7):

(5) $$P=\underset{P_{1}}{\to },\underset{P_{2}}{\to },\text{…},\underset{P_{i}}{\to },\text{…},\underset{P_{n}}{\to }$$

(6) $$P^{\prime }=\overset{\prime }{\underset{P_{1}}{\to }},\overset{\prime }{\underset{P_{2}}{\to }},\text{…},\overset{\prime }{\underset{P_{i}}{\to }},\text{…},\overset{\prime }{\underset{P_{n}}{\to }},$$

where P is the feature point set acquired by the system, $P^{\prime }$ is the feature point set of the reference object, $\underset{P_{i}}{\to }$ is the ith element of the feature point set P, and $\overset{\prime }{\underset{P_{i}}{\to }}$ is the ith element of the feature point set $P^{\prime }$.

(7) $$\underset{P_{i}}{\to }=R\overset{{}^{\prime }}{\underset{P_{i}}{\to }}+\underset{t}{\to }$$

(8) $$\min_{R,\;\underset{t}{\to }}J=\frac{1}{2}\sum_{i\;=\;1}^{n}{\underset{P_{i}}{\to }-\left(R{\underset{P_{i}}{\to }}^{{}^{\prime }}+\underset{t}{\to }\right)}_{2}^{2}\;,$$

where $\underset{P_{i}}{\to }$ is the *i*th element of the feature point set P, $\overset{\prime }{\underset{P_{i}}{\to }}$ is the *i*th element of the feature point set $P^{\prime }$, R is the rotation matrix satisfying the requirements, $\underset{t}{\to }$ is the vector satisfying the requirements, and J is the constructed least‐squares problem.

### 2.5 Isocenter calibration of the 3D virtual model

Isocenter calibration of the 3D virtual model was necessary to ensure that the isocenter of the 3D virtual model was located at the linac isocenter. First, the tumor center, that is, the isocenter of radiotherapy, was determined in TPS, and a virtual cube (16 cm × 16 cm × 16 cm) with the same size as the calibration module (Figure 2a) was created in TPS with the radiotherapy isocenter as the geometric center (Figure 2b). As such, the virtual cube was located in the isocenter of radiotherapy, and 3D modeling was conducted with the patient data as a whole. In clinical operation, the calibration module was first aligned with the room lasers. The 3D virtual model was loaded by AR object recognition and interacted with the 3D virtual model (Figure 2c). The contour information of the calibration module and the virtual cube was processed in real time by an image processing technology, and the distance errors D1 and D2 were calculated (Figure 2d). When D1 and D2 were less than one pixel in three directions, the spatial position of the virtual cube was consistent with that of the calibration module (Figure 2e). The calibration module was removed and the planning target volume (PTV), clinical target volume (CTV), and part of normal tissues were shown (Figure 2f). At this time, the 3D virtual model was the gold standard position for radiotherapy position setup. The technicians could setup patients on the basis of the gold standard position presented by the AR.

### 2.6 LPT and ARPT processes

**FIGURE 3:**
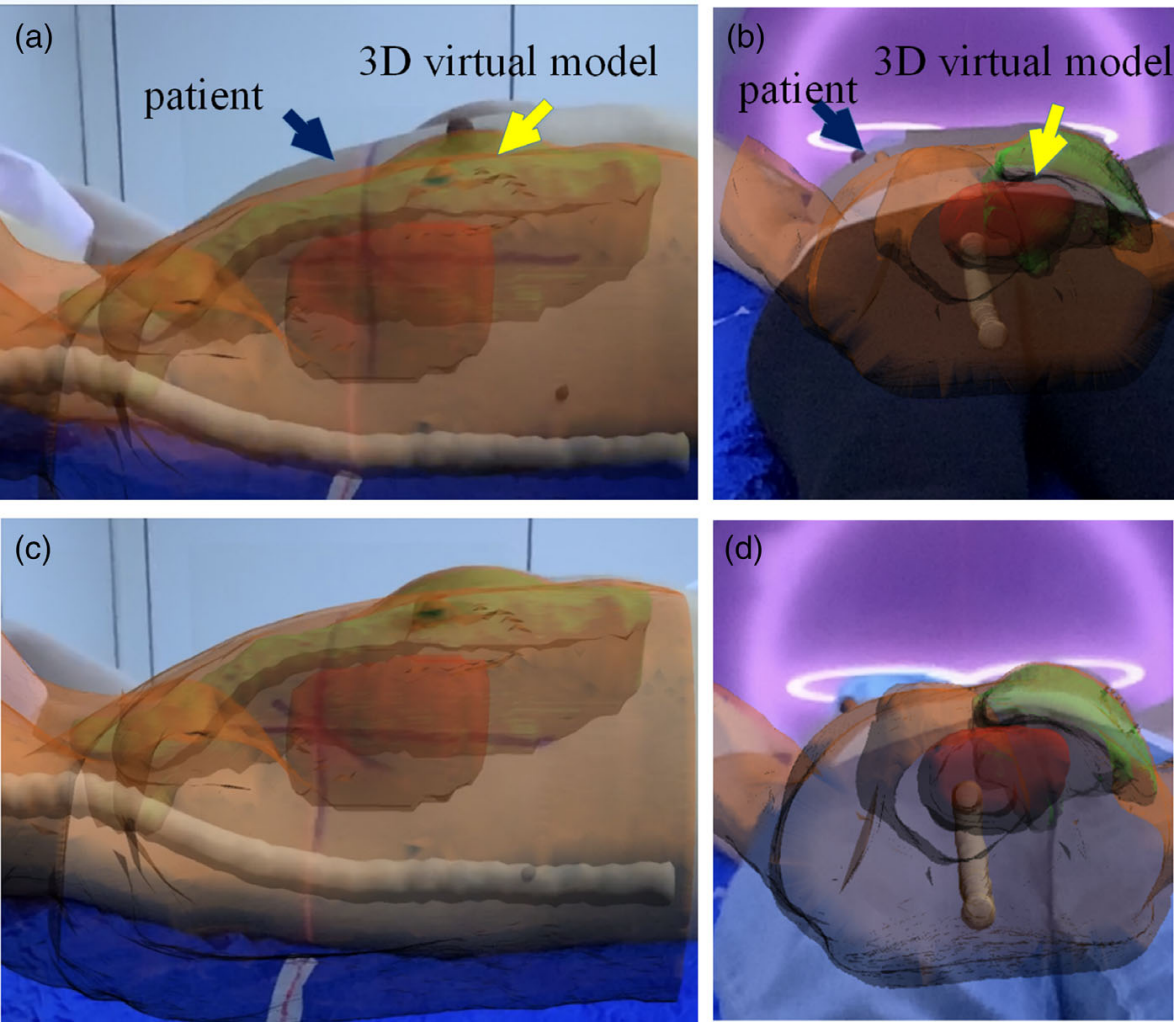
AR position (ARPT) setup procedure (with breast patient as an example): (a) treatment couch side view. The initial position setup is carried out by the conventional laser method, and then the patient is adjusted according to the three‐dimensional (3D) virtual model displayed by AR; (b) treatment couch tail view. The initial position setup is carried out by the conventional laser method, and then the patient is adjusted according to the 3D virtual model displayed by AR; (c) treatment couch side view. The patient is consistent with the 3D virtual model in all directions; (d) treatment couch tail view. The patient is consistent with the 3D virtual model in all directions

**FIGURE 4:**
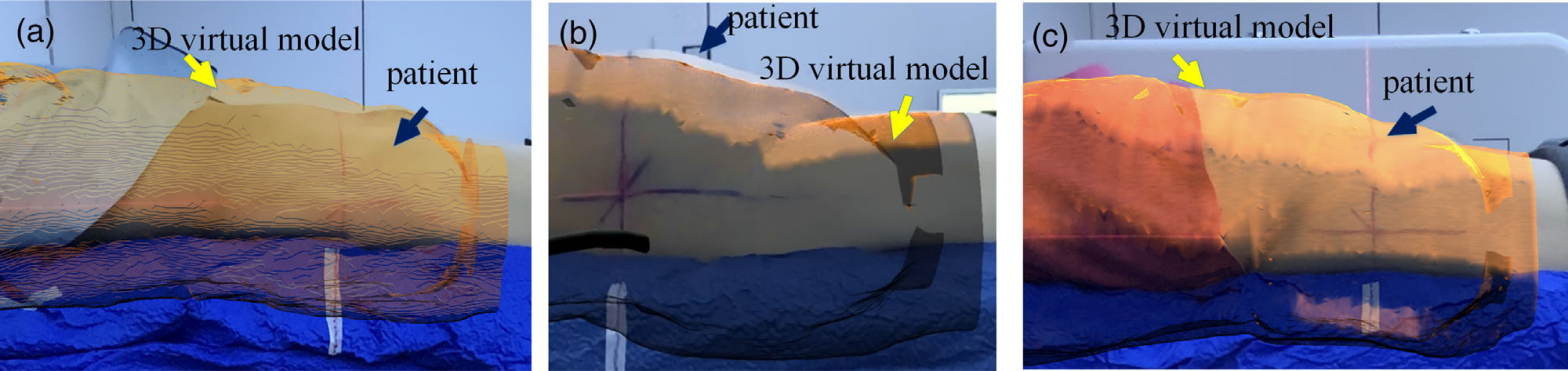
Schematic diagram of bladder filling in abdomen patients: (a) schematic diagram of underfilling of the bladder; (b) schematic diagram of bladder overfilling; (c) schematic diagram of a well‐filled bladder

LPT is mainly based on the alignment of room lasers to the patient's tattoo. In ARPT, the initial position setup was performed by the LPT method, and the 3D virtual model presented by AR was taken as the reference to adjust the patient's position setup (Figure 3a,b). The body surface point cloud $P_{i}$ of the actual patient and the virtual model point cloud $Q_{i}$ were obtained for iterative closest point (ICP) point cloud registration. The translation vector *T* and rotation matrix *R* of all the nearest points between point cloud were calculated to ensure that the patient's position was consistent with the 3D virtual model (Figure 3c,d). CBCT scanning (Elekta, Stockholm, Sweden) was facilitated with tube voltage of 120 kV, scanning speed of 180°/min, frame of 660, frame rotation of 360°, and head and neck, chest and abdomen collimators of M20 and L20, respectively. Then, the ARPT residual error was obtained. Meanwhile, for patients in the abdominal supine position, bladder filling was assessed according to the 3D virtual model (Figure 4). If bladder filling was significantly different from that in simulation CT (Figure 4a,b), radiotherapy was not considered appropriate.

### 2.7 Evaluation metrics

#### 2.7.1 Statistical analysis

SPSS22.0 statistical software was used to process relevant data. The position setup residual errors of each group were evaluated and statistically analyzed using a 95% confidence interval. The Shapiro–Wilk test was used to detect whether the data of each group were normally distributed. Given that all the data in each group conformed to the normal distribution, *t*‐test was used to analyze the differences between ARPT and LPT in each group.

#### 2.7.2 Hausdorff distance

In order to evaluate the coincidence degree between the patient's setup obtained by two kinds of methods and the simulation CT, the Hausdorff distance (HD) was introduced to measure the maximum distance between the point cloud of simulation CT ($P_{s}$) and the point cloud on the patient's surface obtained by CBCT scanning after ARPT and LPT ($P_{c}$), defined as:

$$\mathrm{HD}\left(P_{s},P_{c}\right)=\max\left(\max_{p_{s}\in P_{S}}\min_{p_{c}\in P_{C}}d\left(p_{s},p_{c}\right),\max_{p_{c}\in P_{C}}\min_{p_{s}\in P_{S}}d\left(p_{s},p_{c}\right)\right).$$

However, the maximum HD is susceptible to small outlying regions in $P_{s}$ or $P_{c}$. Therefore, we use 95th and 75th percentile HD (HD95, HD75), which reports the 95 and 75 quantile distance, as more robust measurements in our study.

## 3 RESULTS

A total of 120 sets of position setup residual data of 40 patients were used for error comparison. The LR axis represents the left and right directions, the SI axis represents the superior and inferior directions, and the AP axis represents the anterior and posterior directions, respectively. Rotation represents the deflection angle, pitch represents the pitch angle, and roll represents the roll angle. As shown in Table 1, the G1 and G2 ARPT in all directions of the mean, standard error, and maximum error were less than LPT. Therefore, for patients diagnosed with head and neck cancer, and patients diagnosed with chest and abdomen cancer, ARPT in every axis residual error was smaller and more stable. LPT had a larger residual error and fluctuation.

**TABLE 1:** Translational residual error, rotational residual error, and maximum residual error in different directions in G1 and G2 groups

**Group**	**Method**	**Item**	**LR (mm)**	**SI (mm)**	**AP (mm)**	**Rotation (°)**	**Pitch (°)**	**Roll (°)**
G1	ARPT	$\bar{x}\pm s$	0.68±0.37	1.24±0.37	0.82±0.31	0.48±0.40	0.43±0.32	0.46±0.47
		Max	1.23	1.73	1.07	1.07	0.90	1.13
	LPT	$\bar{x}\pm s$	1.08±0.69	1.38±0.61	1.55±0.57	0.84±0.84	0.87±0.43	0.87±0.56
		Max	1.80	1.77	2.23	2.10	1.23	1.47
G2	ARPT	$\bar{x}\pm s$	1.70±0.74	1.41±0.83	1.64±0.96	0.51±0.48	0.64±0.33	0.50±0.31
		Max	3.10	2.77	4.13	1.27	1.13	0.97
	LPT	$\bar{x}\pm s$	2.19±1.06	3.13±0.85	3.20±1.86	1.54±1.33	1.06±0.54	0.89±0.67
		Max	4.47	4.33	7.73	4.67	1.77	2.40

Abbreviations: AP, anterior and posterior direction; ARPT, AR position setup; LPT, laser position setup; LR, left and right direction; Pitch, pitch angle; Roll, roll angle; Rotation, deflection angle; SI, superior and inferior direction.

As shown in Table 2, for the G1 group, the translational residual error of ARPT in LR, SI, and AP directions in most patients was less than 1 mm. The translational residual error of LPT in LR, SI, and AP directions in most patients was between 1 and 3 mm. For the G2 group, the LPT translation residual error greater than 5 mm occurred frequently in SI and AP directions (16.7% and 21.7%, respectively), and most patients (55% and 51.7%, respectively) had translation residual error greater than 3 mm in SI and AP directions. After ARPT guidance, the patients with translation residual errors greater than 5 mm were reduced from 16.7% to 0% in the SI direction and from 21.7% to 3.3% in the AP direction, respectively, and most of the translation residual errors were controlled within 3 mm.

**TABLE 2:** Error percentage determined by translation residual error threshold (%)

		**LR**		**SI**		**AP**	
**Group**	**Error**	**ARPT**	**LPT**	**ARPT**	**LPT**	**ARPT**	**LPT**
G1	>1 mm	33.3	50	48.3	66.7	50	75
	>3 mm	0	0	0	8.3	0	16.7
G2	>3 mm	13.3	30	16.7	55	16.7	51.7
	>5 mm	0	0	0	16.7	3.3	21.7

Abbreviations: AP, anterior and posterior direction; ARPT, AR position setup; LPT, laser position setup; LR, left and right direction; SI, superior and inferior direction.

As shown in Table 3, most rotation residual errors in G1 and G2 groups were controlled within 1.5°. By adjusting for ARPT, the probability of a rotation residual error greater than 1.5° on the rotation, pitch, and roll axis was significantly reduced in both groups.

**TABLE 3:** Percent of rotation residual error greater than 1.5° (%)

	**Rotation**		**Pitch**		**Roll**	
**Group**	**ARPT**	**LPT**	**ARPT**	**LPT**	**ARPT**	**LPT**
G1	8.3	25	0	33.3	16.7	33.3
G2	5	41.7	3.3	36.7	0	23.3

Abbreviations: ARPT, AR position setup; LPT, laser position setup; Pitch, pitch angle; Roll, roll angle; Rotation, deflection angle.

As shown in Table 4, the difference in translation and rotation residual errors between ARPT and LPT in the six‐dimensional direction in the G1 group was not statistically significant (*P* > 0.05). In the G2 group, the difference in translation residual error in the LR direction was not statistically significant (*P* = 0.218 > 0.05), but that in other directions was significant (*P* < 0.05).

**TABLE 4:** Paired *t*‐test was performed by two position setup methods in G1 and G2 groups

**Group**	**Item**	**LR (mm)**	**SI (mm)**	**AP (mm)**	**Rotation (°)**	**Pitch (°)**	**Roll (°)**
G1	T	−0.88	−0.44	−2.42	−1.58	−1.92	−1.26
	P	0.445	0.687	0.094	0.213	0.151	0.296
G2	T	−1.31	−5.80	−4.98	−2.99	−3.22	−2.29
	P	0.218	0.000	0.000	0.012	0.008	0.043

Abbreviations: AP, anterior and posterior direction; LR, left and right direction; Pitch, pitch angle; Roll, roll angle; Rotation, deflection angle; SI, superior and inferior direction.

Table 5 shows the mean HD95 and HD75 values between the point cloud of the simulation CT and the point cloud on the patient's surface obtained by CBCT scanning after ARPT and LPT to measure the coincidence degree between the patient's setups obtained with two kinds of methods and the simulation CT setup. As seen from Table 5, groups G1 and G2, the mean of HD95 values obtained by ARPT were 4.617 and 5.583 mm, respectively, and those obtained by LPT were 6.821 and 7.292 mm, respectively. The mean of HD75 values obtained by ARPT were 2.613 and 3.201 mm, respectively, and those obtained by LPT were 3.454 and 5.087 mm. The coincidence degree of the patient's setups after ARPT and simulation CT was significantly higher than that after LPT.

**TABLE 5:** Mean of HD95 and HD75values for ARPT and LPT in G1 and G2 groups (mm)

	**HD95**		**HD75**	
**Group**	**ARPT**	**LPT**	**ARPT**	**LPT**
G1	4.617	6.821	2.613	3.454
G2	5.583	7.292	3.201	5.087

Abbreviations: ARPT, AR position setup; HD75, 75th percentile Hausdorff distance; HD95, 95th percentile Hausdorff distance; LPT, laser position setup.

The residual error of ARPT was smaller than that of LPT, especially for patients diagnosed with chest and abdomen cancer.

## 4 DISCUSSION

This study designed an AR‐guided radiotherapy position setup system, and the system could reproduce the patient's posture and position of simulation CT. The system is non‐invasive, non‐radiative, and provides a certain guiding function for radiotherapy position setup. Through experiments, this method was proven as feasible in radiotherapy position setup and could effectively reduce the position setup error and prevented treatment errors.

In 2008, Talbot^15^, ^16^, ^17^ et al. proposed the application of AR in radiotherapy for the first time. A system for visual guidance in the patient's setup for external‐beam radiotherapy procedures was developed by using AR. In this system, a 3D model of the patient's external surface obtained from planning CT data was superimposed onto the treatment couch in the camera images. The augmented monitor could then be viewed, and alignment could be performed against the virtual contour. In 2021, Tarutani^18^ et al. conducted a similar experiment, the main difference being that HoloLens glasses, instead of a camera, were used to complete AR. First, Talbot, J used an ARToolKit package and digital camera to complete the presentation of the 3D virtual model by tracking 20 marks on the surface. Once these marks were blocked, the posture of the presented 3D virtual model changed immediately, and the stability of the 3D virtual model presentation was poor. Moreover, after registration, the system could no longer apply transformations to update the position of the virtual patient contour on‐screen. If any camera movement occurred, the augmentation remained fixed in place on the monitor like an object that was attached directly to the camera lens. In our study, an unmarked AR system was adopted. This system used ARKit software package to call multiple motion sensors such as gyroscopes, accelerometers, and LiDAR sensors, in iPads to build a spatial map of the surrounding environment to realize real‐time tracking and location of the real world. Therefore, it could locate an existing object without additional marks. When the iPad moved, even if the angle of view changed, the relative position between the 3D virtual model and the real environment did not change, and stability and mobility were be guaranteed to some extent. Second, in the previous studies mentioned above, the actual patient and the 3D virtual model were matched only by vision. Such an approach has certain visual subjectivity. In our study, the point cloud of the patient's body surface was acquired and registered with the point cloud of the 3D virtual model, and the patient's setup was constantly adjusted until the average point cloud error with the 3D virtual model was less than 3 mm. In 2014, French^19^ proposed combining AR with a breast radiotherapy position setup that used a breast gelatin model to simulate the availability of AR‐guiding positioning in patients. The RAD‐AR system developed by Cosemtino^20^ in 2017 showed remarkable potential in the application of AR in radiotherapy. However, due to technical limitations, the technologies used in these works were demonstrated only with a RANDO phantom setup but were not applied in the actual radiotherapy process and could not reconstruct the information of important target areas, such as PTV. In our study, in addition to the patient's external surface, the PTV, OAR and other important target areas for radiotherapy were reconstructed, and their relative positional relationship was retained. When AR was used to guide the patient's position setup, the technicians could focus specifically on the body surface setup wherein PTV and OAR were located in addition to the overall alignment. Moreover, in our study, the error data on the AR‐guided position setup and conventional LPT were collected for 240 times in 40 patients with different treatment sites. This approach not only verified the feasibility of AR application in radiotherapy but also showed the application effect of patients with different sites and enabled the systematically analysis of the reasons and clinical significance of different effects.

**FIGURE 5:**
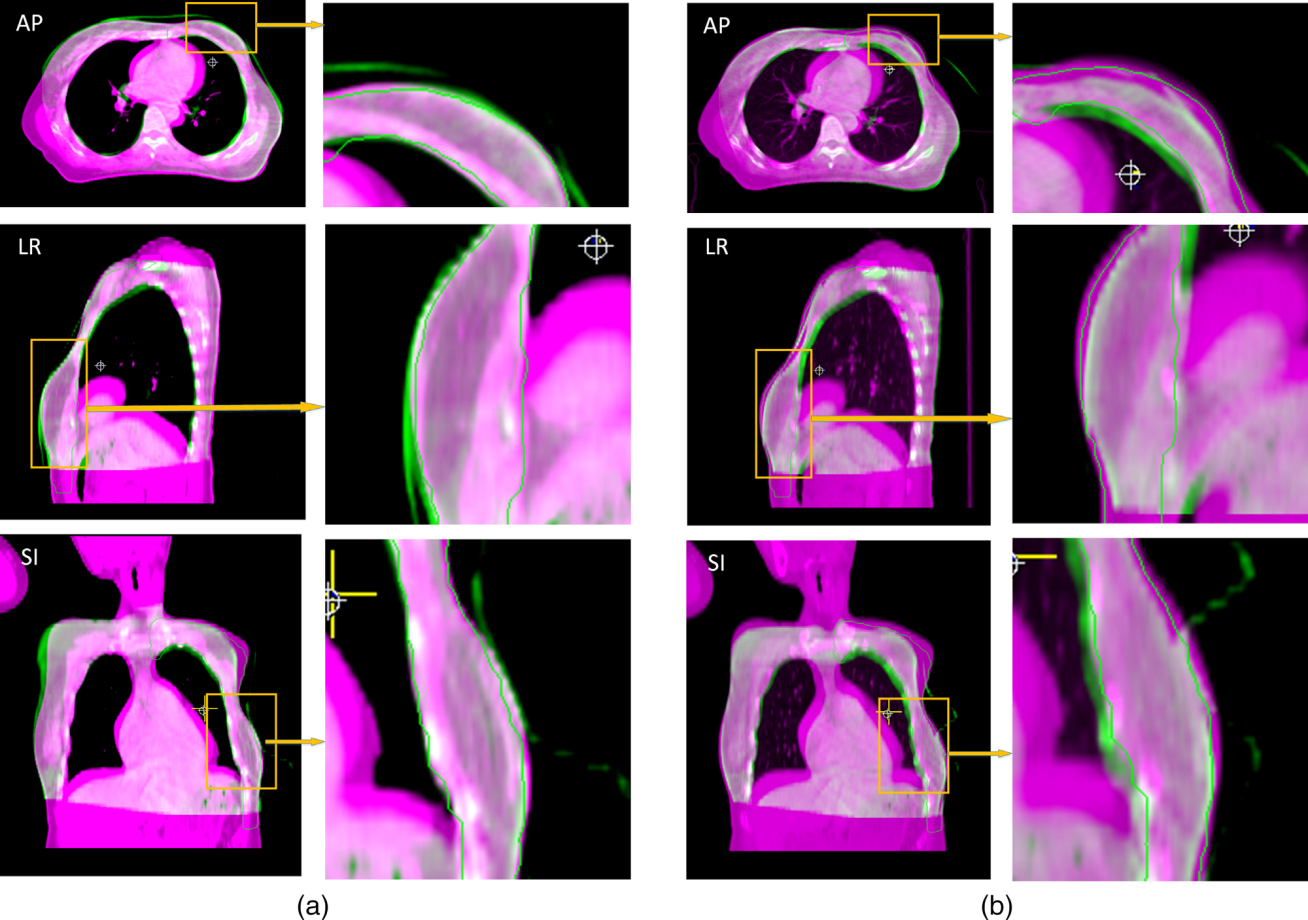
Comparison between the cone beam CT (CBCT) and simulation CT of the same two‐dimensional (2D) CT slice for planning target volume (PTV) in the AP, LR, and SI directions (with breast patient as an example). In the figure, the purple part is the simulation CT image, the green part is the CBCT image of the patient after position setup, and the PTV area is contoured with green lines. (a) AR positioning (ARPT) compared with simulation CT. (b) Laser position setup (LPT) compared with simulation CT

Figure 5 shows the 2D comparison for PTV in the AP, LR, and SI directions between CBCT and simulation CT in the same CT slice after ARPT and LPT. As seen from Figure 5 that in the AP, LR, and SI directions, the CBCT images after ARPT provided a high degree of coincidence with the simulation CT images, whereas the CBCT images after LPT had a poor degree of coincidence with the simulation CT images.

Table 4 shows that the residual error of ARPT compared with LPT for patients diagnosed with head and neck cancer was not significantly reduced. Similar to the results of Zhang^21^ on the optical positioning system for head patients, the translation residual error of head and neck was concentrated in approximately 1 to 2 mm. Tables 1 and 2 show that the residual errors of ARPT in AP and rotation directions were significantly smaller than those of the LPT. After AR guidance, the distribution rate of SI and AP directions greater than 3 mm and the distribution rate of rotation and pitch directions greater than 1.5° all decreased significantly. Thus, ARPT can significantly reduce the number of large errors in the head position setup.

The conventional method of positioning chest and abdomen patients in the supine position is mainly based on the alignment of the patient's tattoo and room laser.^22^ However, the position setup accuracy of radiotherapy is seriously affected when a patient's skin is slack, the tattoo line is blurred, and the skin is pulled^22^ during the position setup. The breast and other non‐rigid body tissues have high mobility^23^ and are prone to morphological changes in the relative position of the limb during fractionated radiotherapy.^24^, ^25^ Thus, positioning patients with plump breasts using skin tattoos is challenging.^24^ The degree of filling of the abdominal bladder directly affects the location of skin^26^ tattoos, tumors, and internal organs.^27^ Jhingran^28^ et al. studied 24 patients and compared the filling and emptying states of the bladder at the time of CT simulation scan. The results showed that the median difference between the maximum and minimum bladder volume was 247 cm^3^, and the vaginal markers placed before and after movement were 0.59, 1.46, and 1.2 cm in the direction of LR, SI, and AP, respectively. Therefore, positioning patients only by the tattoos will result in a large setup error, especially in the direction of SI and AP. The ARPT adopted in our study can reproduce the position and posture of patients intuitively in a wide range, and the body parts’ position out of beam fields will also affect the accuracy of the position setup.^25^ Figure 3 shows a guide on limb placement to ensure the consistency of the relative position and reduce the errors caused by the morphological changes of soft tissues. Figure 4 shows that for abdominal patients with the supine position, the bladder state (over or underfilled) can be determined by observing the position of the skin above the bladder vertically.^29^ Thus, the errors caused by different degrees of bladder filling can be reduced.

In our study, AR was applied in patients’ radiotherapy position setup. ARPT had a better positioning effect than LPT and could reconstruct information on various target areas and allow the technicians to position patients in accordance with the simulation CT posture. In future study, deep learning, neural network, and other contents should be integrated into the AR‐guided radiotherapy position setup system to gradually realize its intelligence and multi‐function and further develop the real‐time monitoring function during radiotherapy.

## 5 CONCLUSION

This study shows that AR‐guided radiotherapy position setup is better than conventional LPT overall, especially for patients diagnosed with chest and abdomen cancer and can effectively reduce the probability of large position setup errors for patients diagnosed with head and neck cancer, which has clinical application value.

## ETHICAL STATEMENT

This study was approved by the Ethics Committee of Changzhou Second People's Hospital Affiliated to Nanjing Medical University (Ethics Number: [2020]KY147‐01), and all patients signed informed consent before radiotherapy.

## CONFLICT OF INTEREST

The authors declare that there is no conflict of interest that could be perceived as prejudicing the impartiality of the research reported.

## AUTHOR CONTRIBUTIONS

The manuscript was written through the contributions of all authors. Chunying Li and Xinye Ni participated in the design of the study, carried out the study, performed the statistical analysis, and drafted the manuscript. Zhengda Lu, Mu He, Jianfeng Sui, and Tao Lin helped to carried out the study. Kai Xie and JiaWei Sun reviewed and edited the manuscript. All authors read and approved the final manuscript.
